# An eye-tracking based robotic scrub nurse: proof of concept

**DOI:** 10.1007/s00464-021-08569-w

**Published:** 2021-06-08

**Authors:** Ahmed Ezzat, Alexandros Kogkas, Josephine Holt, Rudrik Thakkar, Ara Darzi, George Mylonas

**Affiliations:** 1 Hamlyn Centre for Robotic Surgery, Imperial College London, London, SW7 2AZ UK; 2 Department of Surgery and Cancer, Faculty of Medicine, Imperial College London, London, SW7 2AZ UK; 3 University College London, London, UK; 4 St George’s, University of London, London, UK

**Keywords:** Surgery, Robotic scrub nurse, Eye-tracking, Assistive robotic devices, Gaze interactions, Smart operating room

## Abstract

**Background:**

Within surgery, assistive robotic devices (ARD) have reported improved patient outcomes. ARD can offer the surgical team a “third hand” to perform wider tasks and more degrees of motion in comparison with conventional laparoscopy. We test an eye-tracking based robotic scrub nurse (RSN) in a simulated operating room based on a novel real-time framework for theatre-wide 3D gaze localization in a mobile fashion.

**Methods:**

Surgeons performed segmental resection of pig colon and handsewn end-to-end anastomosis while wearing eye-tracking glasses (ETG) assisted by distributed RGB-D motion sensors. To select instruments, surgeons (ST) fixed their gaze on a screen, initiating the RSN to pick up and transfer the item. Comparison was made between the task with the assistance of a human scrub nurse (HSNt) versus the task with the assistance of robotic and human scrub nurse (R&HSNt). Task load (NASA-TLX), technology acceptance (Van der Laan’s), metric data on performance and team communication were measured.

**Results:**

Overall, 10 ST participated. NASA-TLX feedback for ST on HSNt vs R&HSNt usage revealed no significant difference in mental, physical or temporal demands and no change in task performance. ST reported significantly higher frustration score with R&HSNt. Van der Laan’s scores showed positive usefulness and satisfaction scores in using the RSN. No significant difference in operating time was observed.

**Conclusions:**

We report initial findings of our eye-tracking based RSN. This enables mobile, unrestricted hands-free human–robot interaction intra-operatively. Importantly, this platform is deemed non-inferior to HSNt and accepted by ST and HSN test users.

**Supplementary Information:**

The online version contains supplementary material available at 10.1007/s00464-021-08569-w.

Within laparoscopic surgery, robotic devices have been developed to improve clinical outcomes, in so consolidating the shifts towards minimally invasive surgery (MIS). The first marketed surgical robot was the voice controlled laparoscopic camera holder (AESOP). Since then a new era of robotics emerged [1]. The da Vinci® (Intuitive Surgical, Inc.), first emerged in 1997, is a slave robotic manipulator controlled via a computer console by a master-surgeon. To date da Vinci® reports an excess of 1.5 million laparoscopic surgeries, demonstrating reduced post-operative pain, hospital stay and improved surgical accessibility and view in confined anatomical spaces [2]. Such findings have encouraged research of more sophisticated assistive robotic devices (ARD) during surgery. ARD in surgery describes machinery that is controlled by the surgeon in support of surgical task delivery. Recently, the United Kingdom has seen new legislation towards artificial intelligence funding to establish wider ARD within surgery supported by strong evidence [3, 4]. ARD has been hypothesized as an approach to disrupt preventable healthcare human errors, described as one of the main culprits resulting in patient harm [5].

ARD afford surgical teams’ touchless interaction, enhanced information accessibility and task execution; this is apparent in ad-hoc intra-operative retrieval of patient notes or radiological images [6]. From a surgeon’s perspective, ARD may be a “third hand”, thereby allowing the performance of a wider breadth of tasks. Gestix is one example of an automated system which enables the surgeon touchless electronic patient record navigation. The surgeon is able to access imaging using their hand gesture intra-operatively. Hand gesture is captured through a 2D Canon VC-C4 camera mounted on top of a flat screen monitor, in so designating predetermined hand gestures respective functions such as replacing or magnifying the image [7]. In laparoscopic surgery using an ARD, such as the da Vinci® surgical robot, gives the surgeon seven degrees of freedom (DoF) compared to conventional four DoF; this represents the same range of a human wrist in open surgery [8]. ARD can also play a role to improve staff and patient safety, workflow and overall team performance. An example of automated laparoscopic devices initiated by the surgeon’s head pose has been reported by Nhayoung Hong et al., which consequently triggers an endoscopic control system with four DoF to move, in so achieving the desired operative field of view. The system reports 92% accuracy, a short system response time of 0.72 s and a 10% shortening in task completion [9]. HERMES voice recognition interface (VRI) enables pre-determined voice initiated commands during laparoscopic cholecystectomy. The surgeon is able to remotely activate the laparoscopic camera and light source, insufflator to desired intra-abdominal pressure, and switch off all the equipment. One hundred patients were randomized into HERMES assisted surgery and standard laparoscopic surgery. Overall, the HERMES VRI assisted surgery showed significant reduction in completion time across all outcomes measured [10].

Robotic scrub nurses (RSN) support the surgeon in selecting and delivering surgical instruments. The Gestonurse is a magnetic RSN based on surgeon hand gestures which demonstrated 95% accuracy in trials, whilst Penelope is described as a semi-autonomous system based on verbal commands and machine learning [11]. Penelope developers report capability of desired instrument prediction, selection and delivery [11]. These are encouraging but limited by the practicality of disruptive hand gestures and failures in voice recognition when scrubbed in noisy operating theatres [12]. Consequently, there is a need to explore the feasibility of all sensory modalities, in turn enhancing the functionality of future RSN, to enable the surgeon an array of choice during user-RSN interaction.

Within this study, we introduce a novel perceptually-enabled smart operating room concept, based on gaze controlled RSN [13]. This allows the surgeon unrestricted mobility, as naturally occurring intra-operatively [14]. Not only can gaze be tested for its use as a sensory modality to execute RSN tasks intra-operatively, but the integration of eye tracking glasses (ETG) offer the additional advantage over other sensory based interaction of being able to measure real-time surgeon visual behavior. In turn, correlations can be made with intra-operative surgeon mental workload, concentration and fatigue via standard measures including blink rate, gaze drift, and pupillary dilatation [15, 16]. This interface enables dynamic gaze-based surgeon interaction with the RSN to facilitate practical streamlined human–computer interaction in the hope to improve workflow efficiency, patient and staff safety and address assistant shortages.

We report on the usability and acceptability of our RSN and explore their impact on intra-operative communication taxonomy.

## Materials and methods

### Ethics

Study ethics approval was granted by the Imperial College Research Ethics Committee (ICREC) reference 18IC4745.

### System overview

**Fig. 1:**
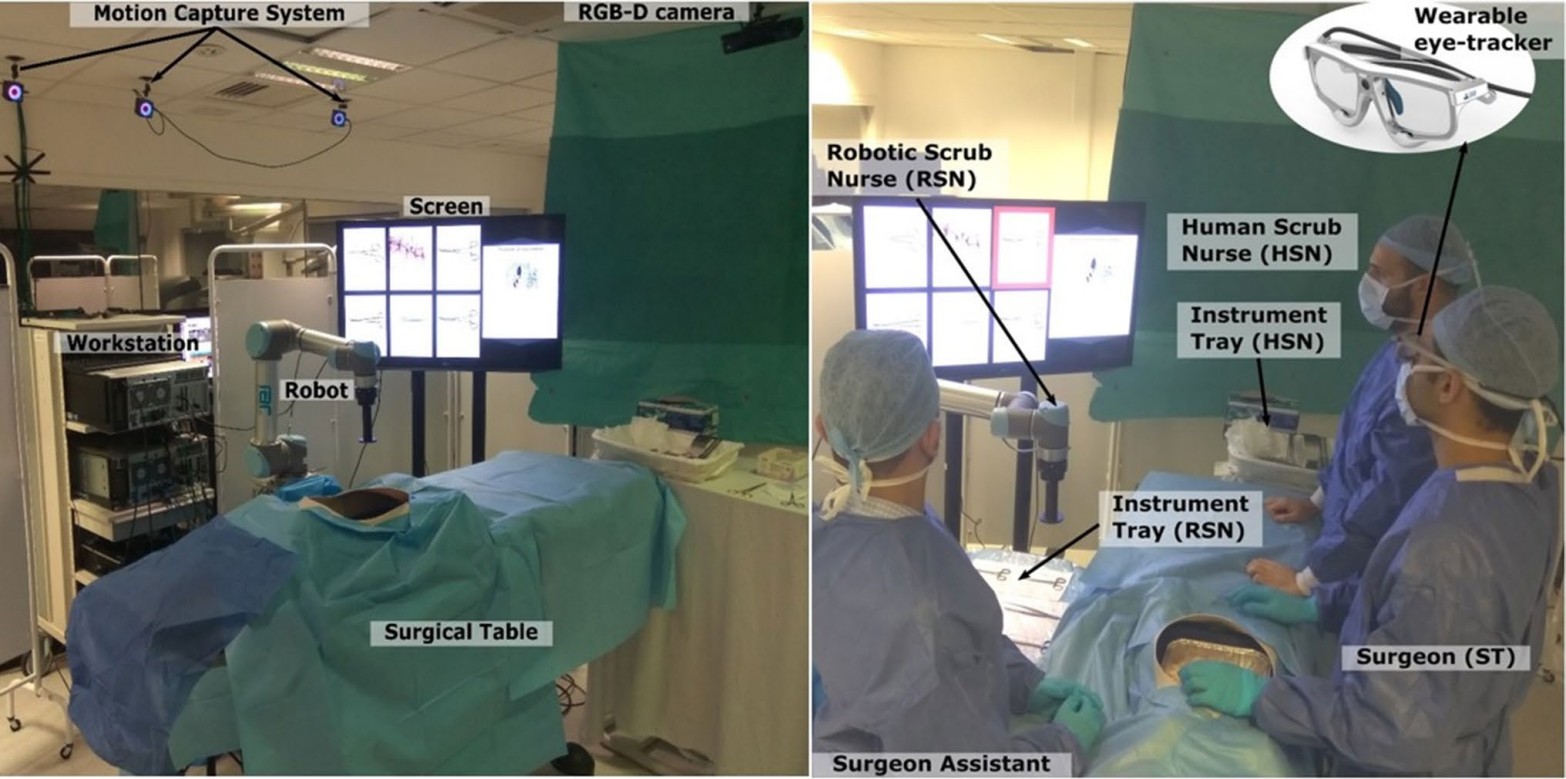
Demonstrating the operative set up. The motion capture system cameras track the spherical markers on the wearable eye-tracker (ETG). The RGB-D cameras provide the 3D model of the operating theatre, in which the user’s 3D gaze is localized. The surgeon (ST) gazes on the screen to select an instrument and the robotic scrub nurse RSN delivers it. The surgeon assistant assists with the surgical task and returns the used instruments to the RSN tray. The human scrub nurse (HSN) delivers instruments from a different instrument tray

System functionality relies on the user’s 3D point of regard (PoR), provided by a real-time framework developed by Kogkas et al. [13, 14]. The user wears an eye tracker, resembling framed glasses (Fig. 1). The pose of the ETG scene camera is estimated in a world coordinate system (WCS). The scene camera pose is equivalent to the user’s head pose, and the gaze ray provided by the ETG helps map fixations to 3D points in the WCS. The WCS is defined by multiple co-registered RGB-D sensors and a motion capture system. These are depth sensing devices integrated with an RGB camera. The 3D fixation, combined with parameters retrieved by an off-line calibration routine, yields the user’s fixation information on a screen. Finally, a graphical user interface (GUI) on the screen guides the user to gaze-controlled instrument selection, which in turn is delivered by an articulated robot.

### Equipment

For eye-tracking, the SMI Eye Tracking Glasses 2 Wireless (SMI ETG 2w, SensoMotoric Instruments GmbH) are used. For RGB-D sensing, the Microsoft Kinect for Windows v2 time-of-flight camera (30 Hz, field of view—FoV of depth sensing 70° × 60°, operating distances 0.5–4.5 m) and for head pose tracking the OptiTrack motion capture system (NaturalPoint, Inc.) is used, with four Prime 13 cameras (240 fps, FoV 42° × 56°). The robot arm is a UR5 (Universal Robots A/S), a 6 DoF collaborative robot with a reach radius of up to 850 mm, maximum 5 kg pay-load and weighing 18.4 kg. It has the Robotiq FT-300 force-torque mounted on its end-effector. For the instrument selection GUI, a 42′′ LG screen is used.

### Offline calibration

Eye fixations were mapped to the ETG’s scene camera via a calibration routine, where users fixate at 9 pre-determined points in the scene camera’s FoV, while keeping their head pose fixed. The position of the surgical instruments in respect to the robot is defined by manually moving the end-effector towards each instrument and recording the target pose. Instruments are intentionally positioned on the tray with corresponding instrument images.

### Interface design

**Fig. 2:**
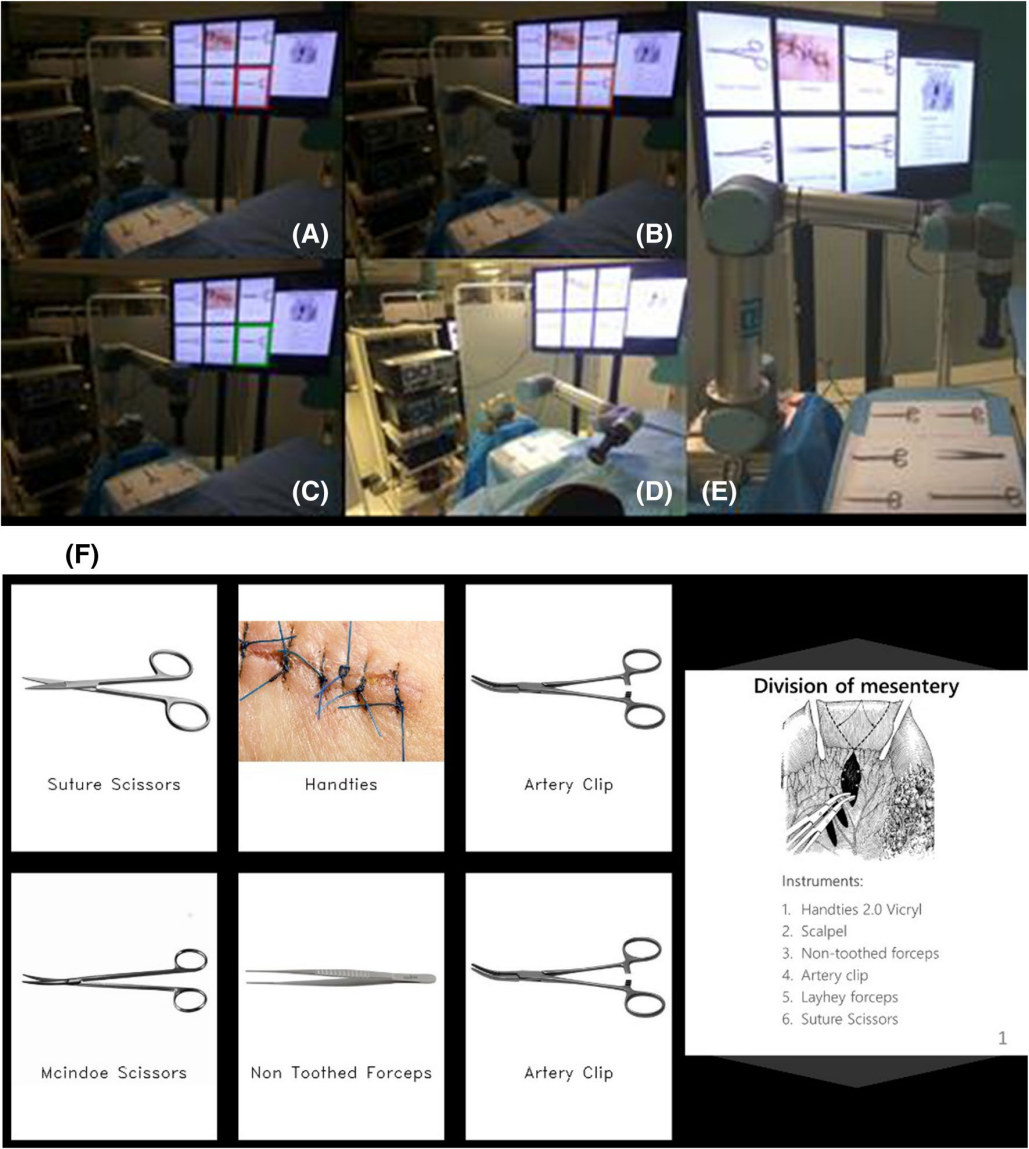
**A**–**D** Egocentric view of the surgical instrument selection routine. **A** The surgical trainee (surgeon—ST) looks at an instrument (red), **B** the instrument is preselected (orange), **C** then selected (green) and **D** the robot delivers it to the ST. **E** Instrument tray, robotic scrub nurse and screen demonstrating the inventory of surgical instruments and main stages of the task. **F** ST screen view of the surgical instruments (left 2/3) and the main operative stages (right 1/3). ST is able to use their gaze to replace the slide with another to view the next step (Color figure online)

The GUI displayed on the screen consists of two parts: instrument selection (left 2/3 of the screen) and the image navigation (right 1/3) as shown in Fig. 2. Six designated blocks equally split demonstrate surgical instruments. When the user visually fixates on any block during instrument selection, a traffic light sequence (red-amber-green) initiates, followed by audio feedback. Starting with red block borders, dwell time of 0.6 s into the same block turns the borders into orange, then a further 1 s turns them into green. The interface is based on pilot experiments and provides audible and visual feedback for the detected fixation on an instrument block (red), signaling before final selection (amber) and action confirmation (green). The time intervals are decided based on a balance between avoiding the Midas touch problem (unintentional gaze-based selection) and disrupting task workflow. As shown in Fig. 2 right, three slides are presented to provide task workflow relevant information. The user can navigate through the slides by fixating on the top and bottom 1/6 parts of the screen for previous and next slide respectively. Dwell time here is 1 s.

### Application workflow

The user wearing the ETG is able to roam freely. A traffic light selection sequence is triggered when fixation on a block is detected on the screen. The robot attaches to the selected stainless-steel instrument via a magnetic gripper on its end effector and delivers it to the user. Following user instrument collection, which is sensed by the F/T sensor mounted on the robot end effector, the robot returns to a pre-configured stationary position.

### Experimental setup and task

Surgeons (ST) were recruited to perform ex vivo resection of a pig colon and hand sewn end-to-end anastomosis. Each surgeon performed two experiments in randomized order:

- A Human scrub nurse only task (HSNt) with the assistance of a human scrub nurse (HSN).
- A Robot and human scrub nurse task (R&HSNt) with the assistance of both RSN and HSN.

Each procedure duration for each participant was estimated at 1 h, although no time restriction was introduced by the research team. Six relevant instruments were considered and assigned to a RSN instrument tray. All instruments were made of stainless steel. These included standard surgical instruments: a non-toothed (*DeBakey*) forceps, curved (*Mcindoe*) scissors, suture scissors, two surgical (mosquito) clips and a 2.0 *vicryl* suture on a surgical (mosquito) clip. The main stages of the task are presented on the right part of the screen (Fig. 2).

For the R&HSNt, the ST performs offline ETG calibration for 1 min. During the task, the surgeon looks at the screen to select the instrument. Once collected from the RSN and used, an assistant surgeon is prompted to or instinctively returns the instrument to its tray position.

ST verbally communicates with the HSN for more instruments and vocally indicates if a wrong instrument is delivered. If eye-tracking recalibration is necessary (due to inadvertent and considerable movement of the ETG), the task continues afterwards. During the HSNt the setup is identical without the screen or RSN. ST communicates with the HSN to deliver instruments. The ETG is utilized to capture and analyze visual behavior.

During both experiments, distractions are introduced to the HSN. A scrub nurse assistant asks the HSN to stop and perform an instrument count twice and solve a cognitive puzzle at specific stages; start (dissection of mesentery), middle (formation of posterior wall bowel anastomosis), end (formation of anterior wall bowel anastomosis).

After each task, the ST and HSN completed *NASA-TLX* and Van der Laan’s technology acceptance questionnaires to compare perspectives of both groups (ST and HSN) on both experiments (HSNt and R&HSNt).

### Participant recruitment

Participants were recruited voluntarily and could withdraw at any stage from the study. STs with normal and corrected vision (wearing glasses) were included in recruitment. Only participants aged 18 or older were included. All HSN included were exclusively theatre scrub nurses in their usual day to day nursing role. At the time of our study design, three groups of up to 20 participants based on their surgical experience would be evaluated; these are junior surgeons with 3–4 years surgical experience, middle grade surgeons with 5–7 years of experience and expert surgeons who had completed their surgical training such as fellows and consultants. Inclusion criteria consisted of STs who were specialist surgical registrars with a minimum of 3 years of surgical registrar experience. All surgeons included were novel to gaze based ARD.

### Subjective validation

#### Task load

After each task, the ST and HSN were asked to complete a NASA-TLX (System Task Load Index defined by NASA) questionnaire. The scale assesses the mental, physical and temporal demand, own performance, frustration levels and effort during the task. An overall task load score is calculated as described in [15].

#### Technology acceptance

Technology usability and satisfaction feedback was collected immediately following the R&HSNt using the Van Der Laan acceptance scale [16]. The scale consists of five usefulness metrics (useful/useless, good/bad, effective/superfluous, assisting/worthless, raising alertness/sleep-inducing) and four satisfaction metrics (pleasant/unpleasant, nice/annoying, likeable/irritating, desirable/undesirable). Each item was on answered a 5-point semantic differential from − 2 to + 2.

### Objective validation

#### Workflow metrics

Performance was assessed in terms of overall *task completion time*. The task starts with the surgeon assistant’s oral instruction “START'” and finishes with the oral indication “FINISH”.

*Workflow interruptions* were measured for both tasks. Interruptions were defined in the HSNt as the events of a wrong instrument delivery by the HSN and the interruption of the task by the ST for > 3 s waiting for instrument delivery. During the R&HSNt, the HSN interruptions and RSN-related events are measured, such as incorrect delivery of instruments and eye-tracking recalibrations.

*Instrument delivery times* were measured for both tasks (Table 2). For HSN this refers to the interval between ST verbal commands to HSN delivery. For RSN it is defined as the interval between the moment the ST starts gazing on the screen to locate the instrument, until the robot delivers the instrument.

#### Visual behavior

Eye gaze data were collected during the experiments. Analysis of the metrics related to task load, attention and fatigue was conducted, namely fixations and pupil diameter.

#### Verbal communication

Verbal communication was observed through videos recorded during the experiments. A new verbal encounter is where there was silence for more than 3 s or a change in the type of communication classified as task, social or gratitude related communication.

### Data analysis

The comparisons demonstrated in the following sections were conducted using within-subjects analysis when comparing:

- Task completion time of HSNt vs R&HSNt
- Number of interruptions in HSNt vs R&HSNt
- NASA-TLX scores of ST in HSNt vs R&HSNt
- NASA-TLX scores of HSN in HSNt vs R&HSNt
- Gaze behavior metrics in HSNt vs R&HSNt
- Instruments delivery time of HSN in HSNt vs R&HSNt
- Verbal communication metrics in HSNt vs R&HSNt

Between-subjects analysis was conducted when comparing:

- NASA-TLX scores of HSNt by ST vs HSN
- NASA-TLX scores of R&HSNt by ST vs HSN
- Van der Laan’s scores by ST vs HSN
- Instruments delivery time of HSN vs RSN in R&HSNt
- Instruments delivery time of HSN in HSNt vs RSN in R&HSNt

For within-subjects analysis, the Shapiro–Wilk test for normality of the paired differences was performed, followed by paired-samples *t*-test when the test was successful. The Wilcoxon signed-rank test was used in non-parametric datasets.

For between-subjects analysis, the Shapiro–Wilk test for normality of the samples was performed, followed by independent-samples *t*-test when the test was successful. In case of non-normal distribution of any of the two samples, the Mann–Whitney *U* test was applied.

For all types of statistical analysis tests, a *p*-value < 0.05 was considered significant.

Data was missing for participant 5 due to technical issues during R&HSNt (verbal communications, HSN and RSN instrument delivery times).

## Results

### Participants

Ten ST participated (7 male and 3 female). Two had corrected vision. Recruitment of staff was logistically challenging due to the complexity of multi-disciplinary ST and HSN recruitment. As such middle grade surgical registrars were recruited. Surgeons were between 30 and 40 years with 6 years surgical experience. Five trained theater scrub nurses were recruited. One ST, with 2 years surgical experience, assisted the ST in all experiments. A medical student acted as scrub nurse assistant.

### Task load (NASA-TLX)

**Fig. 3:**
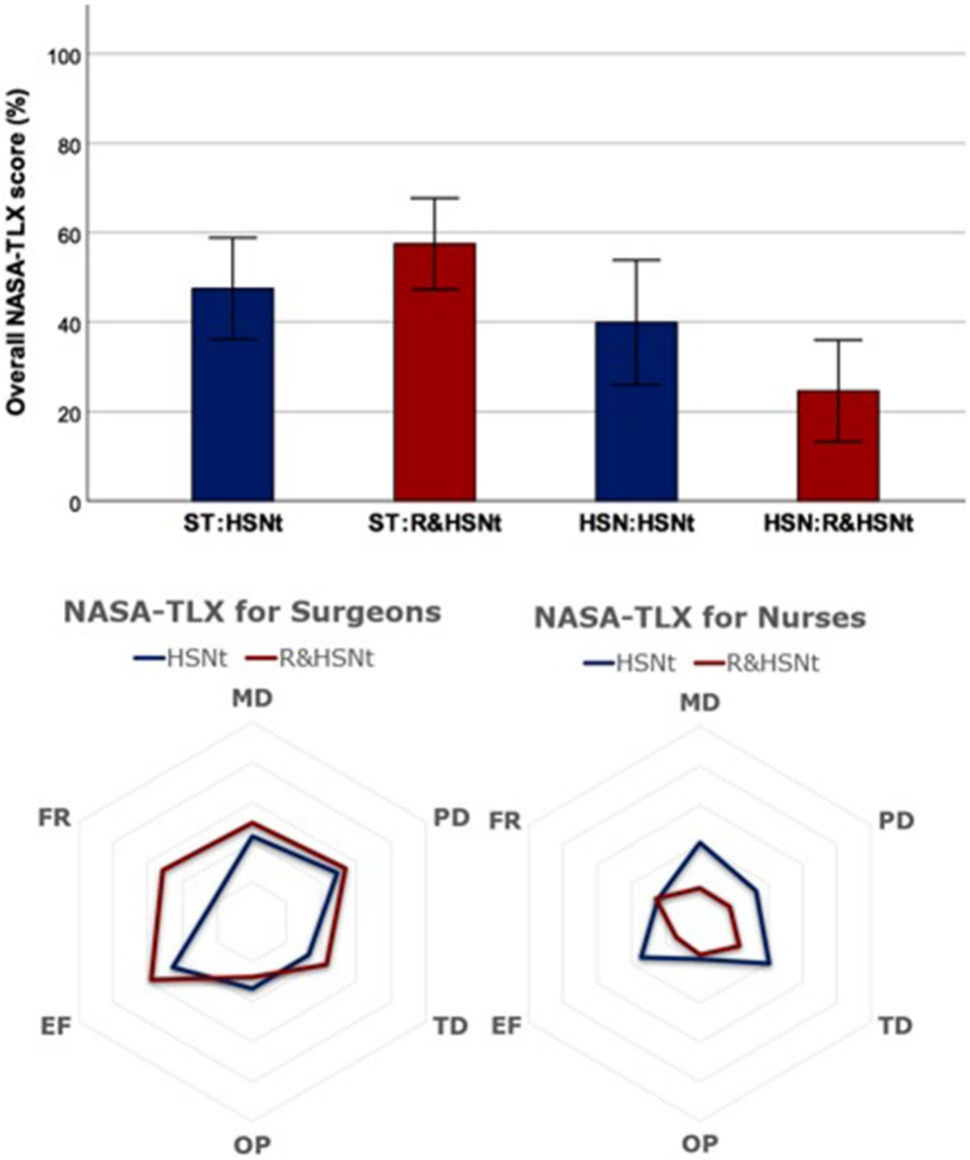
Top: Overall NASA-TLX score and analytical results (*MD* mental demand, *PD* physical demand, *TD* temporal demand, *OP* operator performance, *EF* effect, *FR* frustration level) for (bottom left) Surgeons (ST) and (bottom right) Human scrub nurses (HSN). NASA-TLX values range between 0 and 100, with higher values indicating higher task load (*HSNt* human scrub nurse only task, *R&HSNt* robot and human scrub nurse task)

The NASA-TLX scores are depicted in Fig. 3. ST subjective feedback reported no significant difference overall between HSNt vs R&HSNt (Table 1). ST did not report any significant change on task performance (*p* = 0.526). ST did report significant frustration using RSN, 22 ± 10.6 HSNt vs 51.5 ± 19.3 R&HSNt, *p* = 0.012.

**Table 1:** NASA-TLX comparison of ST and HSN on HSNt and R&HSNt

	ST on: HSNt vs R&HSNt	HSN on: HSNt vs R&HSNt	HSNt by: ST vs HSN	R&HSNt by: ST vs HSN
MD	0.309	**0**.**008**	0.858	**0**.**004**
PD	0.812	**0**.**026**	0.141	**0**.**001**
TD	0.249	0.081	0.451	**0**.**020**
OP	0.526	0.657	**0**.**023**	**0**.**019**
EF	0.120	**0**.**009**	0.147	<** 0**.**001**
FR	**0**.**012**	0.833	0.730	**0**.**017**
Overall	0.052	**0**.**017**	0.161	<** 0**.**001**

*p*-values are reported

*MD* mental demand, *PD* physical demand, *TD* temporal demand, *OP* operator performance, *EF* effort, *FR* frustration, *HSNt* human scrub nurse only task, *R&HSNt* robot and human scrub nurse task, *HSN* human scrub nurse, *RSN* robotic scrub nurse, *ST* surgical trainee

Statistical significance *p* < 0.05 are in bold

Overall, HSN reported significant difference (39.9 ± 19.6 HSNt vs 24.6 ± 15.9 R&HSNt, *p* = 0.017). Frustration remained unchanged (*p* = 0.833), whilst mental, physical demand and effort showed significant differences in favor of the R&HSNt.

Comparison of ST vs HSN using RSN showed significant difference overall (57.5 ± 14.2 vs 24.6 ± 15.9, *p* < 0.001) and specifically in all sub-scales, in so demonstrating reduced HSN demands. Comparison of ST vs HSN perceptions over the HSNt showed no significant difference overall (*p* = 0.161).

### Technology acceptance (Van der Laan)

**Fig. 4:**
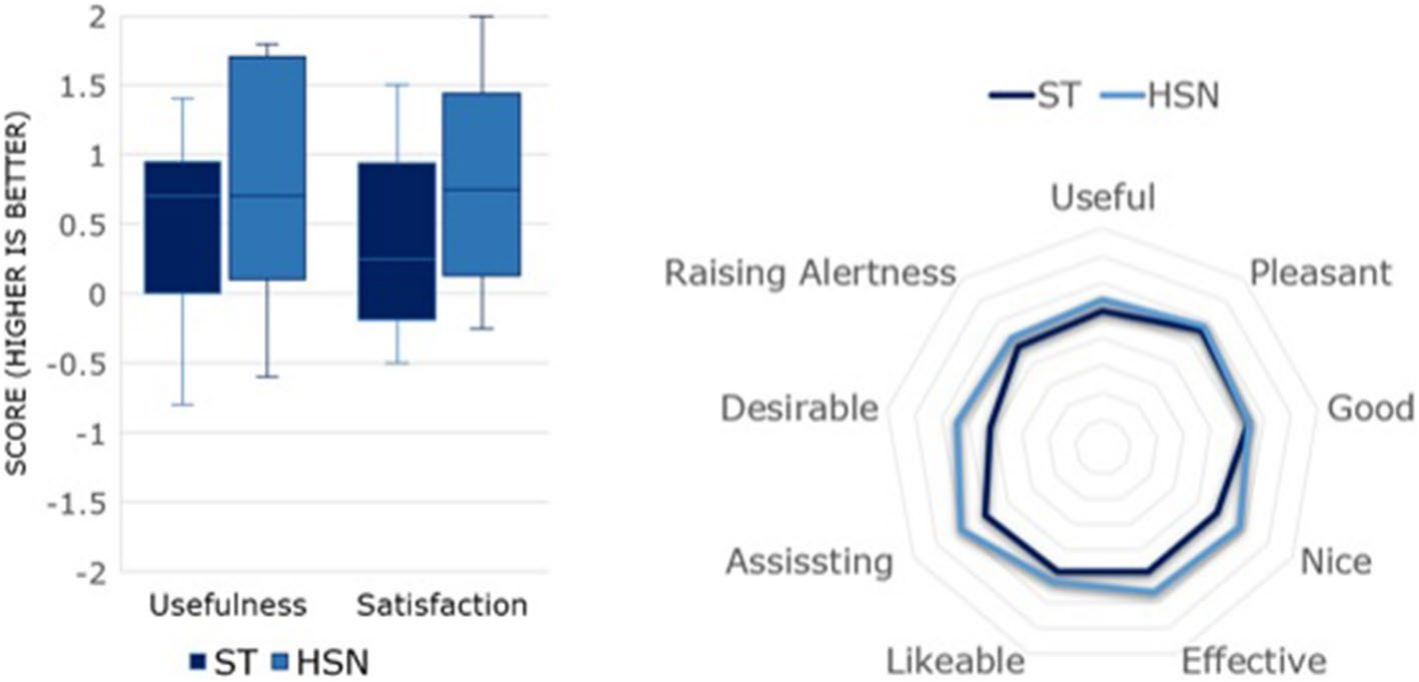
Left: Overall Van der Laan’s technology acceptance score by Surgeons (ST) and Human scrub nurses (HSN) and (right) analytical results. The usefulness scale derives from the average of *useful/useless, good/bad, effective/superfluous, assisting/worthless, raising alertness/sleep-inducing metrics* and satisfaction scale derives from *pleasant/unpleasant, nice/annoying, likeable/irritating, desirable/undesirable* metrics. The scale ranges between − 2 and + 2, with higher values indicating positive bias on the specific attribute

The ST group reported usefulness score of 0.5 ± 0.73 and satisfying score of 0.43 ± 0.74 (Fig. 4). ST reported that the RSN was likable 0.4 ± 0.84, useful 0.5 ± 1.08 and pleasant 0.8 ± 0.79. ST feedback was neutral about RSN desirability 0.1 ± 0.99. HSN feedback reported usefulness score of 0.76 ± 0.92 and satisfying score of 0.78 ± 0.79. HSN reported RSN was likable 0.6 ± 1.26, useful 0.7 ± 1.42 and pleasant 0.9 ± 0.99. RSN was perceived as desirable 0.7 ± 0.82. Upon comparison of ST vs HSN using RSN there was no statistically significant difference in *technology acceptance* domains; *p* = 0.491 for usefulness and 0.32 for satisfaction. Overall responses were positive in ST and HSN groups (usefulness score of 0.5 ± 0.73/satisfying score of 0.43 ± 0.74 vs usefulness score of 0.76 ± 0.92 and satisfying score of 0.78 ± 0.79, respectively).

### Workflow metrics

In summary, mean *task completion time* was 22:35 ± 6:30 [16:02, 37:17] min vs 26:04 ± 4:50 [20:18, 34:35] min (HSNt vs R&HSNt, respectively), *p* = 0.074. This includes R&HSNt ETG recalibration time within the latter experiment.

There was no statistical significance in the total number of *workflow interruptions* per task (*p* = 0.84) between R&HSNt and HSNt (2.3 ± 0.95 vs 2.4 ± 1.26, respectively).

During HSNt, the HSN reported a median *instrument delivery time* of 2.2 s (interquartile range 3.0). In R&HSNt, HSN reported 5.3 s (6.7) and the RSN 6.1 s (3.3). HSN within HSNt was significantly faster compared with HSN and RSN in R&HSNt, and non-significant across HSN and RSN in the R&HSNt.

### Visual behavior

Comparative analysis between the two tasks showed no significant difference in all metrics related to gaze behavior, except for the pupillometry metrics. Fixation rate per second was 2.7 ± 0.46 HSNt vs 2.59 ± 0.62 R&HSNt, *p* = 0.455. Average fixation duration in milliseconds was 250 ± 58 vs 269 ± 69, *p* = 0.298. The average pupil diameter of both eyes during the HSNt (4.26 ± 0.71 mm) is larger than during the R&HSNt (3.74 ± 0.67 mm, *p* < 0.001). It has been shown that pupil dilation is related to increased difficulty with a task and cognitive effort, while decreased pupil diameter may indicate tiredness [17, 18]. However, variations in the brightness of the environment can also produce changes in the pupil size. In our experiments, lighting conditions were kept uniform by using the same lighting, blinding the windows, and using screens around the operating space.

### Verbal communication

There was a statistically significant difference in verbal communication upon comparison, in that HSNt exhibited twice as many verbal communication episodes; task related communication 34.3 HSNt vs 16.8 R&HSNt, *p* = 0.008. There was no significant difference in social or gratitude related communication (Table 3).

### Subjective feedback

The general consensus was positive about the potential of RSN in surgery. All ST reported that fixating on the screen away from the operative view impacted on overall task flow. Seven STs mentioned a combination with verbal based commands would enhance the RSN. An intuitive RSN platform which can learn surgeon selections and predict instruments was raised. Three ST highlighted HSN would supersede an RSN in the event of unpredictable events or emergencies. All HSN were positive in describing the RSN and had no concerns over role replacement.

## Discussion

Our novel eye tracking RSN augments existing modalities in facilitating surgeon-RSN interaction [9, 11]. The surgeon fixates on the desired instrument via a screen, initiating the respective retrieval and delivery by the RSN [13]. Our study addressed gaze-controlled RSN assistance compared against traditional setups of a scrub nurse alone, to identify system usability and limitations.

### Task metrics

Use of gaze demonstrated no significant difference in the overall task completion time (*p* = 0.074), despite longer instrument delivery times in R&HSNt vs HSNt (Table 2). Randomization of sequence of R&HSNt vs HSNt was performed to eliminate learning bias. Similarly, recruited ST were at similar residency training. All RSN tasks were completed, with no significant difference in task interruptions in R&HSNt vs HSNt. The RSN was occasionally interrupted for recalibration. RSN exhibited 100% correct instrument selection rate. HSNt interruptions mostly occurred during instrument count/puzzle solving tasks to simulate intra-operative nurse disruptions. These interruptions included incorrect instrument transfer or instrument transfer delay. An instrument count is protected scrub nurse time to avoid inadvertent loss or retention of surgical instruments inside the patient [19, 20]. These observations are congruous with the reported 3.5% errors in drug administration during nurse task disruption [21]. The disruption may result in cognitive load shifting towards that new task, affecting time to primary task completion or total neglect [22]. This poses a risk within the context of nurse shortages, especially in more complex and longer operative tasks with frequent scrub nurse interruption and instrument demand [23].

**Table 2:** Instrument delivery time (s)

HSNt				R&HSNt							
HSN (1)				HSN (2)				RSN (3)			
Mean	SD	Median	IQR	Mean	SD	Median	IQR	Mean	SD	Median	IQR
3.8	5.4	2.2	3.0	6.3	4.9	5.3	6.7	7.1	4.2	6.1	3.3
(1) vs (2) *p*-value						<** 0**.**001**					
(2) vs (3) *p*-value						0.409					
(1) vs (3) *p*-value						<** 0**.**001**					

Mean, median, standard deviation (SD) and interquartile range (IQR) of all delivery times are reported. *p*-values are reported for comparison of each participants median delivery time

*HSNt* human scrub nurse only task, *R&HSNt* robot and human scrub nurse task, *HSN* human scrub nurse, *RSN* robotic scrub nurse

Statistical significance *p* < 0.05 are in bold

### User metrics

NASA-TLX feedback shows positive perception towards the RSN (Fig. 3). ST and HSN users perceived no significant differences in task performance between HSNt and R&HSNt. This infers non-inferiority of the RSN. ST mental demands in delivering tasks were not significantly different across HSNt vs R&HSNt, suggesting no cognitive overload, linked to negative performance, thus avoidable adverse outcomes [24]. All surgeons were novel to gaze based ARD which may partly explain ST frustration. Qualitative feedback reported interrupting gaze away from the surgical field caused frustration. To negate this, we suggest researchers develop RSN that utilizes a combination of gaze with light see-through wearable displays, hand gesture and voice-based recognition, allowing surgeons the choice between communication modalities, as within conventional surgery. This freedom should address user frustration, improve system practicality and enhance the surgeon’s operative skill development and ability [25]. In our study, we simulated open surgery, whereas in laparoscopic procedures, the operative field is screen-based, hence the RSN utility would be less disruptive. Further studies are planned.

Standardized Van der Laan technology acceptance scores reported positive outcomes across usefulness and satisfaction with HSN group exhibiting higher scores (ST and HSN groups: usefulness score of 0.5 ± 0.73/satisfying score of 0.43 ± 0.74 vs usefulness score of 0.76 ± 0.92 and satisfying score of 0.78 ± 0.79, respectively) (Fig. 4). HSN dismissed fears about ARDs replacing their role, but welcomed their assistance. The RSN enables HSN to perform more complex tasks within major multisystem operations where specialist instrument assemblies, or an “extra hand” is needed.

### Communication taxonomy

Communication intra-operatively impacts on patient safety and takes place between the surgeon and scrub nurse via verbal and non-verbal cues. Non-verbal cues include body language, eye contact, and hand gestures [26]. Researchers report a failure of shared information quality a third of the time, risking patient safety due to cognitive overload and task interruption [27]. The impact of ARD on communication breakdown in surgery, therefore information relay and safe task execution, has been questioned [28]. The author stipulates a structured team-based communication framework would negate any communication breakdown and enhance team usage of ARD [29].

We demonstrate a significant reduction in verbal communication frequency between ST and HSN, accounted by task related communication, where the surgeon asked for an instrument or operative command (Table 3). Gratitude was displayed to HSN following their instrument delivery (mean 5.0 HSNt vs 1.7 R&HSNt). This difference during R&HSNt reflects reduced HSN instrument delivery. The social communication, defined as communication unrelated to task performance, showed no significant difference across HSNt and R&HSNt. Arguably, this communication type enhances team personability thereby improving team dynamics and reducing communication failures [30]. Emerging evidence, during the COVID-19 pandemic, suggests wearing additional personal protective equipment could impede surgeons’ ability to communicate with the surgical teams including the scrub nurse. This may be related to reduced visibility and voice clarity through a filtering surgical mask [31]. In such circumstance, the RSN is an alternative useful adjunct. Additionally, the RSN may limit avoidable staff exposure to infected patients during aerosol generating procedures.

**Table 3:** Mean, standard deviation (SD) and comparison of communication episodes between the human scrub nurse only task (HSNt) and robot & human scrub nurse task (R&HSNt)

	HSNt	R&HSNt	*p*
Task	34.3 [19.0]	16.8 [10.0]	**0**.**008**
Social	11.1 [8.9]	7.1 [6.5]	0.187
Gratitude	5.0 [6.0]	1.7 [2.0]	0.081
Total	50.4 [22.4]	25.6 [15.1]	**0**.**005**

Statistical significance *p* < 0.05 are in bold

### Crew resource management: a shift in paradigm

Crew resource management derives from aviation to describe management structures which optimize available resources including people, processes and equipment to maintain safety and efficiency [32]. One example is the potential risk reduction in sharps injuries during instrument transfer estimated at up to 7% of all surgical procedures [33]. Using the RSN enables the HSN to be more involved in complex surgeries repeatedly, utilizing and reinforcing their experience, causing a paradigm shift in HSN job roles towards “assisting”. Similarly, HSN can become experts in those procedures, enhancing performance and patient outcomes, as adopted across America [34]. We observed the HSN often only responded to verbal communication initiated by the surgeon. This may reflect old hierarchical attitudes leading to a lack of nursing empowerment to raise clinical opinions and challenge their concerns; “Cannot intubate, Cannot ventilate” is an example of this. A young woman admitted for routine surgery could not be intubated. A nurse brought an emergency airway kit for tracheostomy and then informed the anesthetists who dismissed her. Delayed tracheostomy led to fatal brain anoxia [35].

The RSN enables the senior surgeon, in effect the team leader, to adopt a type of hands off leadership, coined Lighthouse Leadership. Lighthouse Leadership is used within cardiac arrest resuscitations. Team leaders only directly intervene when needed, taking a “step back” to observe and plan situations more effectively. This leadership ethos embeds stronger team structures, empowers surgical residents in training, in so significantly improving task performance and resuscitation outcomes [36].

### Limitations and future work

In this study, we report on a gaze based RSN which was tested within an open surgical approach. We accept MIS lends itself as a more natural setting where the surgeon looks at the monitor to operate, in so avoiding any gaze related interruptions during instrument selection. Open surgery was selected within this setting to enable a skilled procedure of intermediate time duration which demanded constant instrument replacement throughout the task. This in turn allowed the testing of the gaze-based screen fixation and instrument selection to demonstrate its reliability when used frequently. In comparison, a MIS such as laparoscopic cholecystectomy would require minimal instrument replacement and would not reflect the usability of the system. Admittedly, we plan future testing in MIS to demonstrate its utility. The RSN system appears physically large but it is mobile and has been positioned as a second surgical assistant; users expressed no concerns regarding the size of impediment in task completion. Another limitation of the study is the requirement of an assistant to return the instruments once delivered by the RSN then used by the ST. We acknowledge this RSN system is a proof of concept for gaze as a modality of interaction in a master/slave interface and further development in the RSN is required to return the instrument to the instrument tray after use. We believe this would greatly enhance its practicality as an independent RSN. We stipulate an RSN system should combine a variety of sensory modalities such as voice, hand gesture and gaze to mimic natural human–human interaction during surgery. This would address user frustration through the use of gaze alone, particularly within open surgery rather than MIS. The use of gaze offers exciting future potential supported by continuous developments in artificial intelligence (AI) and computer vision; this will allow surgical phase recognition and prediction. The use of an RSN in combination with AI could pre-empt surgeon needs of instruments and make them available earlier, while saving this “dead time” for the HSN to use more efficiently.

## Conclusions

In this study, we report on a gaze based RSN which enables the surgeon hands-free interaction and unrestricted movement intra-operatively. Initial findings for proof of concept demonstrate acceptability of RSN by ST and HSN; all participants were novel to this system. Importantly, RSN is shown to be non-inferior to conventional HSN assistance, in that operative completion rate, duration and perceived user task performance were not significantly different. No instrument delivery errors were reported by the RSN. Social communication behaviors amongst staff did not significantly differ intra-operatively. There is particular scope in laparoscopic surgery to use gaze- based RSN, where the ST naturally fixates on the screen, in so reducing RSN related frustration during gaze interruptions observed within open ex-vivo surgery during instrument selection. In the latter, researchers should endeavor in developing an intuitive RSN based on multiple sensory modalities, in so affording the ST the choice in interaction as with human–human interaction. We aim to expand our eye tracking RSN to recognize and track instruments in real-time, enabling workflow segmentation, task phase recognition and task anticipation.

## Supplementary Information

Below is the link to the electronic supplementary material.

Supplementary file1 (MP4 4612 kb)
